# “The Wall Now Between Us”: Teaching Math to Students with Disabilities During the COVID Spring of 2020

**DOI:** 10.1007/s40299-021-00568-8

**Published:** 2021-05-10

**Authors:** Rachel Lambert, Rachel Schuck

**Affiliations:** Gevirtz Graduate School of Education, Department of Education, University of California, 18 Ocean Road, Isla Vista, Santa Barbara, CA 93117 USA

**Keywords:** Special education, Online learning, COVID-19, Students with disabilities, Mathematics education

## Abstract

This paper presents a case study of the experiences of a special educator named Ms. Montes (pseudonym) teaching standards-based mathematics during Emergency Remote Teaching (ERT) during spring 2020. Ms. Montes was interviewed twice during this period; data were analyzed through inductive thematic analysis. Pre-COVID, Ms. Montes provided her students daily opportunities to tackle challenging mathematical problems and taught self-regulation strategies for students to better understand themselves as learners. After the shift to ERT, Ms. Montes described “the wall between us” as various barriers that made teaching mathematics online far more challenging. Challenges included supporting students with productive struggle when not physically present with them and supporting student self-regulation during mathematical problem-solving. Supporting students with disabilities to learn mathematics during ERT and distance learning will require considering emotional and affective dimensions of learning. Coaching students and families in self-regulation strategies could support student engagement in mathematical problem-solving in online learning.

Due to COVID-19, in spring 2020 teachers across the world transitioned to Emergency Remote Teaching (ERT; Hodges et al., 2020). The sudden closure of schools has affected all students, but perhaps none more dramatically than disabled students.^1^ Students with disabilities face additional challenges learning online such as diminished motivation and a lack of accommodations (Serianni & Coy, 2014). Pre-COVID, students with disabilities underperformed in mathematics compared to their nondisabled peers, affecting student opportunity (Wei et al., 2013). As students with disabilities already have diminished access to conceptual and challenging mathematics (e.g. Jackson & Neel, 2006), we are particularly concerned with how special educators engage students with disabilities in the rigorous, problem-based mathematics of the Common Core State Standards of Mathematics (CCSSM, 2010) during the pandemic. Inequities in opportunities to learn mathematics are likely to become more pronounced during ERT. Though some preliminary research has begun to document the challenges facing special educators during this time (Schuck & Lambert, 2020; Tomaino et al., 2020) little is known about how special educators are adapting their teaching in the area of mathematics. This case study documents the shift to ERT for a unique and important case: a special educator skilled in providing mathematics aligned with the CCSSM for her elementary-aged students in a self-contained classroom.

## Literature Review

We review the literature in three areas: (1) motivation in mathematics, (2) mathematical problem-solving for students with disabilities, and (3) online learning for students with disabilities. Motivation is a critical issue in learning mathematics through challenging problem-solving, a key component of mathematics education reforms in the US. We also review previous research in online learning that included students with disabilities. Together, these three areas of previous research provide understanding of the challenges special educators face when teaching mathematics aligned with the CCSS-M during ERT.

### Motivation and Engagement in Mathematics

Motivation, both intrinsic and extrinsic, is central to engagement in mathematics. Motivation can be increased through opportunities for autonomy, as well as curriculum that is relevant and engaging (Ainley & Hidi, 2014). Motivation can also be affected by the emotional valence of mathematics learning; mathematics is the only subject in school with its own anxiety condition (mathematics anxiety) and is emotionally processed (Eligio, 2017).

Middleton et al. (2016) suggest that studies of motivation in mathematics should shift from studying motivation as a long-term emotional state/trait to the study of motivation as in-the-moment engagement in mathematical tasks. Motivation is not just an initial impulse to engage in mathematical tasks, but an ongoing process that involves anticipation and evaluation of the mathematics, as well as strategies to self-regulate, solve problems, and reflect on one’s actions (Zimmerman, 2005). As summarized by Middleton et al, “mathematics engagement involves the simultaneous recruitment of motivational and affective structures to guide sustained, productive learning behavior” (2018, p. 18). Multiple factors such as individual mathematical dispositions and classroom sociomathematical norms interact to produce in-the-moment engagement, which is always influenced by motivation. Engagement can be conceptualized as the interplay of behavioral, cognitive, emotional, and social factors (Rimm-Kaufman et al., 2015).

Motivation is key for online learning success (Hartnett, 2016); therefore, students who do not have high academic motivation may struggle to engage independently (Greer et al., 2014). Motivation is an especially salient challenge for those who struggle with organization and planning, such as students with disabilities (Serianni & Coy, 2014). While Marteney and Bernadowski’s (2016) survey of 80 online teachers of students with disabilities found that nearly 85% felt that student motivation to engage was increased with proper feedback and instruction, provision of such feedback and instruction is not always easy. For example, Rice and Carter (2016) found that online high school teachers struggled to teach self-regulation skills, including motivation, to their students. One potentially effective way to increase motivation in subjects like mathematics where students with disabilities tend to have low confidence is gamification (Wen et al., 2020).

### Mathematical Problem-Solving and Students with Disabilities

The focus of this paper, Ms. Montes (pseudonym), teaches in California, a US state that has adopted the US Common Core State Standards (CCSS; 2010). These standards center mathematical problem-solving rather than memorization. One prominent research tradition that emphasizes problem-based learning is Cognitively Guided Instruction (CGI; Carpenter et al., 2000, 2014). CGI research has documented how students, including young children, can solve complex problems using their own strategies, often using manipulatives or drawing, rather than being taught specific procedures. Emergent research has investigated the beneficial effects of CGI on teachers of students with disabilities (Moscardini, 2014) and on the mathematical learning of students with disabilities (Foote & Lambert, 2011).

Developed using research on the practices of expert mathematical learners, the Standards of Mathematical Practice (SMP) in the CCSS describe how students can strategically engage in complex problem-solving. In order for students to learn in mathematics classrooms centered on problem-solving, students must deeply engage for sustained periods of time and need to be comfortable engaging in *productive struggle*. Productive struggle refers to sustaining student engagement in challenging problem-solving (Warshauer, 2015). Warshauer found that teachers were successful in engaging students in productive struggle through particular teacher moves: acknowledging student struggle, encouraging perseverance, and questioning their mathematical thinking. Teachers of students with disabilities need to allow students to struggle while solving problems so that students can both develop deeper understandings of content and develop perseverance as mathematical problem solvers (Lynch et al., 2018).

Students with disabilities are offered less access to sustained problem-solving, as special education has focused on direct instruction of procedures over open-ended problem-solving (Lambert & Tan, 2020). Similarly, students with disabilities have been offered fewer opportunities to engage in standards-based mathematics in classrooms (Jackson & Neel, 2006; Kurz et al., 2014). In previous work, we have located this problem in a lack of access to mathematical meaning-making caused in part by deficit conceptions of disabled students (Lambert, 2018).

The majority of students in Ms. Montes’ classroom had an Individual Education Program (IEP) for Specific Learning Disability (LD). Students with these disabilities often have difficulties with executive functioning and language processing (Cortiella & Horowitz, 2014). Another persistent issue for these students is the pervasive low expectations throughout their schooling, which may cause students to internalize their difficulties as fixed (Lambert et al., 2019). This may lead to the lower academic motivation seen in students with disabilities (Langberg et al., 2018; Modesto-Lowe et al., 2013; Sideridis & Scanlon, 2006).

### Online Learning for Students with Disabilities Before and During COVID-19

Research conducted prior to the pandemic has identified potential benefits of online learning for students with disabilities; for example, the ability to freely and repeatedly access course materials (Serianni & Coy, 2014) and increased parental awareness of their child’s education (Sorensen, 2019). However, research on online learning for disabled students prior to the COVID-19 pandemic is unlikely to be directly relevant to most teachers and students facing ERT. Nearly all studies of K-12 special education distance learning were conducted with families (and teachers) who knowingly chose online learning over traditional brick-and-mortar schools. Given that ERT was forced upon everyone with little notice, students and families were likely much less motivated (and/or able) to engage than in prior studies. Even if parents are motivated, online learning for young children with disabilities requires high levels of parent involvement (Burdette & Greer, 2014), which can be challenging for parents (Rice et al., 2019; Smith et al., 2016). Given the mental health effects of losing in-person schooling that some students with disabilities may now feel (Lee, 2020; Patel, 2020), along with the fact that many parents may not be able to provide their child with the assistance they need (Schuck & Lambert, 2020), many students may have trouble engaging with online learning. Indeed, almost 10% of parents of non-disabled children interviewed by Garbe et al. (2020) indicated low motivation to engage online was impeding their child’s learning. Preliminary findings from multiple recent studies have shown that, as the period of online learning progresses, student engagement has decreased, both in general education populations (Kim et al., 2020) and amongst those in special education (Balkist & Agustiani, 2020; Smith, 2020).

### Current Study

Few recent studies have gone into detail with regards to what special education teachers are doing to address the decreased engagement and motivation during ERT. Therefore we sought to understand how an experienced elementary-level special educator transferred her teaching online in response to the pandemic. Because motivation is particularly crucial with regards to engaging in mathematical problem-solving (especially for students with disabilities), we decided to highlight mathematics teaching. The focus on the elementary period was important, as it may be particularly challenging to motivate younger children to engage in remote mathematics learning.

Our study aimed to answer the following research questions:

1. What issues emerged in ERT for an experienced special educator in spring 2020?
2. What shifts did this teacher perceive in student engagement in mathematical problem-solving and how did she respond?

## Methods

### Setting and Participants

Three elementary special education teachers participated in this study, though this paper only discusses one of them, Ms. Montes. Discussion of the two teachers who taught students with more significant support needs can be found elsewhere (Schuck & Lambert, 2020). Ms. Montes teaches a 3rd–5th grade classroom at a predominantly Hispanic/Latinx (> 90%) and socioeconomically disadvantaged (> 70%) school, with approximately 18% English language learners and 13% students with disabilities. Ms. Montes teaches a self-contained special education class with primarily students with IEPs for Specific Learning Disabilities, with a few students with IEPs for Autism. Her class during this period included 13 students—3 girls and 10 boys. District personnel named her as an exceptional teacher, particularly in mathematics. She identifies as Latina and has over ten years of experience as a special educator.

#### Ms. Montes’ Mathematics Instruction Pre-ERT

Before this study, Ms. Montes participated in a four-year professional development on Cognitively Guided Instruction (Carpenter et al., 2014) led by the first author. The first author visited Ms. Montes’s classroom four times to observe mathematics. Despite challenges in communication, processing, and executive functioning, students in Ms. Montes’s class solved complex problems without direct instruction, were able to share their thinking, and engaged in the strategic thinking of others. Ms. Montes’s classroom was notable in three ways. First, she held high expectations, both in the rigor of the problems and encouragement of student independence. Second, students had freedom to choose their problem-solving strategies (e.g. students could solve problems alone or in groups, using drawing, manipulatives, or calculators). Third, Ms. Montes focused on developing her students’ strategic self-understandings, so that students would see themselves as mathematics learners and create their own accommodations. Ms. Montes often used FlipGrid on iPads for students to record their strategies in short videos.

### Data Collection and Analysis

The first author, an experienced qualitative interviewer, conducted an individual interview with Ms. Montes approximately two weeks after schools closed that was 26 min long. An interview guide was developed beforehand with pertinent questions (e.g. How many of your students have you been able to reach? How are families dealing with the transition? What educational techniques have you used so far?). The interview took an informal conversational approach (Patton, 2002), beginning with, “How’s it going?” and continuing with follow-up questions to document the teacher’s experience during ERT, particularly with regards to mathematics instruction. The following topics were highlighted: student engagement, family engagement, and shifts in mathematics instruction. A focus group with three special educators teaching mathematics was conducted a month later, with a duration of 1 h. The focus group again used an interview guide and took a conversational tone, focusing on shifts since the first interview. Participants were then presented with the themes from the first interview as a member check.

An inductive approach to analyzing the interview was chosen, as the topic being studied was so novel that no existing coding scheme applied (Miles et al., 2020). Both authors read the interview transcript and generated initial codes. Together, these initial codes were discussed and compared. We took our consensus codes and went through the transcript again using focused coding (Bogdan & Biklan, 1997). This second round of coding was first completed separately and then together, resolving discrepancies. A memo summarizing the themes generated from the interview was used in a focus group with all three participants held one month later. Both authors coded Ms. Montes’ answers from the focus group using the themes generated from her first interview. New themes were discussed via consensus and integrated into analytic memos. Ms. Montes was consulted for another member check after the completion of the first draft of findings.

## Findings

Our analysis of Ms. Montes’ interviews generated four themes: Inequitable Access, Socio-Emotional Focused Teaching, Supporting Self-Regulation, and Tensions in Planning Instruction. Throughout our presentation of the themes, we keep in mind our research questions regarding both general issues that emerged in ERT and issues regarding engagement in mathematical problem-solving.

### Inequitable Access

As school closed in spring 2020, Ms. Montes immediately attempted to make contact with all her students. Many caregivers had to work during school hours. One child stayed with an aunt, as his mother was concerned that she could not provide appropriate support for at-home learning since the mother was not fluent in English, even though Ms. Montes was bilingual. Ms. Montes used this as an example of how disruptive the current situation was for families. Conscious of how challenging the situation was, Ms. Montes repeatedly stated her intention to make sure her demands were not making an already tense situation worse for families.

There was wide variation in the amount of support students were given at home. Some students had an adult working alongside them all day; others did not receive any one-on-one assistance. Some students did not engage in online learning due to lack of support, while other students engaged with little or no adult support. Ms. Montes had a few students who seemed to thrive in distance learning, including one who consistently asked her for advanced problems. For this student, Ms. Montes noted that school at home seemed to mean less distraction and anxiety.

### Socio-Emotional Focused Teaching

As she began holding synchronous Zoom meetings a few weeks after school shut down, Ms. Montes described beginning with socio-emotional learning. She let her students dictate the content of these early Zoom meetings, allowing them to check-in with each other:

The kids wanted to talk to each other. It was hilarious. In one screen, you see somebody having their little cars zooming by, and another one, somebody is holding up like five Pokémon cards, just switching through them. And another one, somebody's got like a family picture. And I'm just like, ‘You guys, you're supposed to be listening!’ But they were just so excited to see each other. So we were just doing share-outs.

Ms. Montes soon added academic content to Zoom meetings:

By the end of the week, it was more academic. And I lost two students because of that; they have not returned to Zoom, once they realized it wasn't just chatting time… It's concerning, because I can't get hold of those families.

Ms. Montes also discussed how her students’ socio-emotional connections to each other affected her mathematics teaching. In her in-person classroom, students would support each other, checking-in to see how each other was doing. This was now lost during ERT: “All of a sudden, for mathematics, there's no one to turn and say, ‘Are you doing this today? Are you getting this? What are you going to do?’” Ms. Montes identified peer discussion as a critical support for engagement, which can lead to both finding the correct answer and fostering a sense of community.

### Supporting Self-regulation and Independence

Ms. Montes had a pronounced focus on what she called “independence.” Her classroom had an explicit focus on developing self-regulation: “Not only do we work on academic stuff at school, we're working on that emotional self-regulation, just self-monitoring, what routines look like.” However, she soon saw that this kind of independence was not transferring from the classroom to home:

In school, I feel like they have this certain sense of autonomy, like there's an expected autonomy and expected ‘I can do this for myself.’ And right now at home, I'm seeing a lot of, ‘Well, no, at home, I can't do stuff by myself. At home, there's people that will either do things for me or tell me when I do it wrong.’

One family member attended all of their child’s Zoom meetings. Another student’s work changed significantly since the transition, likely because of overzealous adult support.

On top of being overinvolved, some parents were not implementing the kind of self-regulation strategies Ms. Montes had taught her students. She mentioned a “brilliant” student who she had taught to independently take breaks pre-closure:

The wall right now between us is: I can't provide that structure that he needs… He needs constant emotional check-in. And mom called me one day in tears because she's like, ‘He's really hard to work with.’ And I'm like, ‘Yeah,’ I'm like, ‘But he needs a break.’ ‘So why does he need breaks? He's so far behind, he should get less breaks.’ And I'm like, ‘No, no, it's okay.’ Also, he's home, so it's fine! Those are the walls…. Our kids need structure so badly.

Even when some students were attempting to implement the strategies they had learned in class, parents were not always supportive:

Some of my kids who are independent, and I've taught them how to self-regulate and take their own breaks when they need it. The parents don't trust them. They're like, ‘Are they supposed to be taking this many breaks? How long is the break supposed to be?’ And I'm just like, it's okay.

Ms. Montes did not blame parents for this disconnect but analyzed the situation as a failure of communication on her part: “That's been like a big eye opener as far as a good thing: how much I need to make parents aware that I really work on independent skills.” She saw this as an opportunity to reflect on how to communicate the importance of these skills to parents throughout the school year.

For Ms. Montes, the prospect of letting her students engage in productive struggle when she was not there to support them was unsettling, as she knew mathematics could lead to “meltdowns” without proper support. Therefore, her strategy during ERT mathematics was to avoid the emotional reactions that she knew were possible: “It’s like, I don't want them to be crying at home. Like at school, they get really frustrated, but I'm there, and I can catch it right before it gets to that point.”

### Tensions in Planning Instruction

Guidance from the school district for spring 2020 was that teachers should focus on consolidating prior knowledge for students with disabilities rather than teach new content. Ms. Montes noted, “The biggest message we're getting is: it's about maintaining and making sure they don't regress.” Ms. Montes struggled with this guidance to simply maintain current skills:

At first I was thinking, am I continuing to push them along? Or am I just making sure they keep what they've had this far?… We were towards the finish line, so we have done a lot already. But I still had a few more things that I wanted to get to. And so how much is it fair for me to try that?

She believed that she needed to push her students into new, challenging content, but also struggled with the pedagogical implications of assigning challenging mathematics when she was not present. The first mathematics instruction that the students experienced during ERT were take-home packets, which only a few students completed. As the spring progressed, Ms. Montes focused on replicating routines that she used in class: giving story problems, using Flipgrid to record solutions, and number talks. These familiar routines did not always go well during ERT:

A lot of them really aren't into FlipGrid from home the way they have been at school. So, I'm just like, what is happening? And so we were doing number talks on Zoom, twice a week. And those also didn't go so great, because all of a sudden, they're all super shy. They don't really want to respond. So I'm just like, what am I going to do?

Some students also appeared to be regressing to less sophisticated mathematical strategies, even when the work seemed similar to work they had done in class, for example during Counting Collections (Franke et al., 2018). In one problem, students were given an image of 84 items and asked to count them, documenting their strategies:

One of my kids, his equation was literally one plus one. And he had all 84 ones in there. And I was just like, that is not what we have been doing in school. It should have at least been 10 plus 10 plus 10… Like we've been doing that all year. Like I've seen you do it in class. But he's one who needs a lot of reassurance. So I think the ones who need reassurance always go back to the ones if there's nobody to tell them, ‘You are a mathematician. You're fine.’

This student had been regularly using base-ten groups to count in school yet returned to counting by ones during ERT.

During the focus group, Ms. Montes described a new weekly routine for mathematics—a set of problems focused around a single story. Since pets were a strong student interest in this class, the first week’s set of problems were all connected to the story of a bunny. Ms. Montes created the story by recording herself reading the problems aloud, using a SnapChat filter to give herself bunny ears:

So then last week, I just did day-by-day math, and I kind of turned it into a whole story… it has a whole narrative and on each slide, I recorded myself reading the slide to give more access to it, because I was wondering if that was part of the problem too – the kids who need to hear it a few times, especially because their reading levels are all over the place. And then I kind of read it in a specific way, pausing in certain places to give them time to visualize the problem.

Ms. Montes noticed a big difference when she structured mathematics this way: “I felt like I got way more success this week. …And I think they really like the bunny’s story, because each day was building off of that bunny.” The personal touch (videos of her reading the problems pretending to be the bunny), consistency around the context, and her efforts at increasing accessibility (reading the problem aloud) seemed to result in more motivation and engagement for the students. As Ms. Montes discussed next steps in her mathematics curriculum, motivation was at the forefront of her planning. She recalled a previous year in which she had taught volume through Minecraft and planned on repeating that unit, with Minecraft as “the motivator.”

## Discussion

This study documents the shift to ERT during the COVID-19 pandemic for a special educator experienced in teaching mathematics through problem-based learning. Analysis of Ms. Montes’ interviews generated four themes related to her instruction during spring of 2020: Inequitable Access, Socio-Emotional Focused Teaching, Supporting Self-Regulation, and Tensions in Planning Instruction. The first two themes are particularly relevant to our first research question asking what issues emerged during ERT for an experienced special educator. The second research question on engagement in mathematical problem-solving is directly addressed in the last two themes: Supporting Self-Regulation and Tensions in Planning Instruction.

### General Issues Faced During ERT

The themes of Inequitable Access and Socio-Emotional Focused Teaching reflect the difficulties Ms. Montes had even attempting to provide her students with academic content. First, some students had trouble even logging in to online classes, a finding that has unfortunately been documented by other COVID-19 researchers (e.g. Garbe et al., 2020; Kim et al., 2020; Schuck & Lambert, 2020). For others, the issue was not necessarily lack of access to technology, but not having an adult available for one-on-one support during the school day. Ms. Montes reacted to this by ensuring that families were not overwhelmed, focusing on providing socio-emotional care before academics-focused instruction. She recognized that one of the major issues with ERT was not just difficulties in accessing curriculum, but that students were missing out on social connections with their peers. Ms. Montes’ experience suggests it may be more difficult for students to engage academically online (as opposed to in-person) because they lack social connectedness. Her emphasis on supporting students’ social development is an important lesson for other teachers who are continuing to teach online.

Though Ms. Montes often connected self-regulation with mathematics (see below for a discussion of this), she also brought up important considerations regarding the role of families in overall self-regulation. Because she was not sitting right next to students, ready to “catch them,” Ms. Montes had to rely on parents to implement coaching around academic self-regulation, a role with which they were not familiar. When instruction was suddenly shifted to home, she noticed that families appeared to assume that students could not perform at grade-level and described over-involved family members who did not give the children space to solve problems. This echoes findings from other special educators teaching during ERT (Schuck & Lambert, 2020) and is perhaps what some of the educators in Tomaino et al. (2020) study were referring to when they implied parents were “interfering” in online learning.

Ms. Montes did not blame parents for not promoting self-regulation and independence. Instead, she planned to provide support such that families could better understand their child’s self-regulation strategies. This finding has implications for future online learning for students with disabilities. Much of the literature on online learning for students with disabilities refers to parents as “learning coaches” (Burdette & Greer, 2014). However, this seems to most often refer to parents coaching students through the curriculum, not through meta-cognitive, self-regulative strategies. Ms. Montes’ desire to teach families about self-regulation strategies also has relevance to in-person special education. Though parents of students with disabilities may want more information from teachers about strategies to implement at home (Azad et al., 2018), it is not clear that they are aware of the importance of self-regulation strategies. It is of the utmost importance for special educators now, during the pandemic, and after, to communicate clearly with parents about the significance of fostering independence and self-regulation.

### Encouraging Engagement in Online Mathematics Instruction

While inequitable access to reliable online learning was a concern for Ms. Montes, she also had concerns about the students who *were* able to log on, particularly with regards to their engagement. Ms. Montes noted a large decrease in engagement in mathematics, which contrasted sharply with her typical in-person classroom. She connected this lack of motivation and engagement with difficulties in self-regulation.

These factors support the theories of mathematical motivation put forward by Middleton et al. (2016), who define mathematical motivation as complex. Here, multiple factors interrelate; motivation is both individual and social. The structure of engagement for these students shifted from the social environment of the classroom to more individual, at-home learning. Mathematics through distance learning, at least initially, became dependent on individual and intrinsic motivation. Intrinsic motivation was more challenging in the online environment for most of Ms. Montes’ students, a finding that is in line with other emergent research on ERT for disabled students (Balkist & Agustiani, 2020; Smith, 2020). In a classroom in which mathematics was a social, peer-intensive event, something was lost in the translation to the digital realm. While a few students seemed more engaged in this context, the majority lost motivation to engage. She saw a shift in engagement when she created problems with contexts that spanned a week, such as the set of problems focused on bunnies. We note that multiple factors may have contributed to the increased motivation of students: the consistent context, attempts at humor and connection to student interests, as well as the multimodal presentation of content (reading problems aloud). These findings are critical as educators work to increase motivation in online mathematics learning.

The second major finding relates to the connections between self-regulation and productive struggle. Motivation can be conceptualized as engagement in in-the-moment mathematics activity including starting the problem-solving process and sustaining engagement through strategic emotional self-regulation (Zimmerman, 2005). Ms. Montes saw a dramatic decrease in students’ use of strategic self-regulation strategies while engaged in learning at home, and she connected self-regulation explicitly to engagement in sustaining mathematical problem-solving through productive struggle.

Previous research on productive struggle has focused on teachers’ cognitive interventions, such as questioning students about their strategies (Lynch et al., 2018; Warshauer, 2015). However, Ms. Montes returned repeatedly to the idea that to persevere through mathematical challenges, her students needed both cognitive *and* affective coaching. She noted that her students, without support, can have “meltdowns” when faced with mathematical challenges, and her role is to “catch it right before they feel hopeless or defeated.” She recognized that productive struggle can become unproductive if students lose confidence or become overwhelmed by the emotions of mathematics (Eligio, 2017).

Teachers can feel an impulse to protect students, particularly those with disabilities, from this kind of struggle. Ms. Montes instead worked to develop students’ cognitive and emotional strategies to persist through this kind of challenge. She stated, “I watch and swoop in with a guiding question that will steer them back on track or with a suggestion about taking a mental break for our brains to regain energy.” The first strategy is cognitive, supported by research on teacher moves to support productive struggle (Warshauer, 2015). The second emphasizes self-regulation and meta-cognition, suggesting a break and making it clear *why* a break will support learning. The virtual wall of ERT made it difficult for Ms. Montes to facilitate self-regulation strategies online, a finding similar to that of Rice and Carter (2016) with regards to online high school teachers.

### Limitations

While this study contributes a detailed account of teaching students with disabilities during ERT, as well as highlights the difficulty of enhancing motivation to engage in mathematics online, it must be viewed within the confines of several limitations. First, as with all qualitative inquiry, we did not aim to produce results that would be generalizable across all educators or even all special educators. Additionally, we were unfortunately unable to observe Ms. Montes’ online classroom directly, nor were we able to include any student perspectives in this study. In fact, we were unable to find any research on online learning that interviewed students with disabilities about their experiences during ERT. Students are a critical source of data on engagement (Rimm-Kaufman et al., 2015), and research must be done to ensure their voices are heard.

## Conclusion

Our analysis of interviews with Ms. Montes revealed several challenges regarding teaching special education during the beginning stages of the COVID-19 school closures. She recognized the systemic issues that impeded her students’ ability to engage (e.g. lack of access to technology, adults who were not always available to act as “learning coaches”), as well as the difficulties of encouraging motivation in students in an online setting, particularly with regards to mathematics. She highlighted the importance of socio-emotional connectedness and self-regulation in mathematics, and eventually began to find ways to creatively present mathematics in a way that mirrored her rigorous in-person classroom. While she acknowledged the challenge of expecting parents to work on these self-regulation skills, she saw this as a learning opportunity to build stronger home-school relationships in the future.

### Implications and Future Research

Supporting students with disabilities to learn mathematics during ERT and distance learning will require considering emotional and affective dimensions of learning. These considerations are necessary to provide conditions within which students can engage in mathematics. Coaching students in self-regulation strategies can support them in engaging in productive struggle in mathematics. Interventions that do not account for emotional factors could result in compounding educational trauma and diminished motivation. Educators should support families in not only the academic progress of their students, but their development of self-regulation strategies. As Ms. Montes suggested, these kinds of strategies are critical for work at home and should be a regular part of family-school communication.

Future research in this area could explore how a larger sample of special education teachers support students in mathematical problem-solving. Other research could explore ways to support self-regulation across home and school. Particularly, we suggest research that is able to take student perspectives into account. Only through sustained inquiry on the perspectives of teachers, families, and students with disabilities on online learning can we identify the “walls between us” and work to dismantle them.

## Footnotes

1 We use both person-first and identity-first language to describe disability to respect varied preferences in the disability community.
